# Hormonal therapy with estradiol and drospirenone improves endothelium-dependent vasodilation in the coronary bed of ovariectomized spontaneously hypertensive rats

**DOI:** 10.1590/1414-431X20154655

**Published:** 2015-11-17

**Authors:** M.V. Borgo, E.R.G. Claudio, F.B. Silva, W.G. Romero, S.A. Gouvea, M.R. Moysés, R.L. Santos, S.A. Almeida, P.L. Podratz, J.B. Graceli, G.R. Abreu

**Affiliations:** 1 Departamento de Ciências Fisiológicas, Centro de Ciências da Saúde, Universidade Federal de Espírito Santo, Vitória, ES, Brasil; 2 Departamento de Morfologia, Centro de Ciências da Saúde, Universidade Federal do Espírito Santo, Vitória, ES, Brasil

**Keywords:** Menopause, Hypertension, Hormone therapy, Drospirenone, Coronary reactivity

## Abstract

Drospirenone (DRSP) is a progestin with anti-aldosterone properties and it reduces blood pressure in hypertensive women. However, the effects of DRSP on endothelium-dependent coronary vasodilation have not been evaluated. This study investigated the effects of combined therapy with estrogen (E2) and DRSP on endothelium-dependent vasodilation of the coronary bed of ovariectomized (OVX) spontaneously hypertensive rats. Female spontaneously hypertensive rats (n=87) at 12 weeks of age were randomly divided into sham operated (Sham), OVX, OVX treated with E2 (E2), and OVX treated with E2 and DRSP (E2+DRSP) groups. Hemodynamic parameters were directly evaluated by catheter insertion into the femoral artery. Endothelium-dependent vasodilation in response to bradykinin in the coronary arterial bed was assessed using isolated hearts according to a modified Langendorff method. Coronary protein expression of endothelial nitric oxide synthase and estrogen receptor alpha (ER-α) was assessed by Western blotting. Histological slices of coronary arteries were stained with hematoxylin and eosin, and morphometric parameters were analyzed. Oxidative stress was assessed *in situ* by dihydroethidium fluorescence. Ovariectomy increased systolic blood pressure, which was only prevented by E2+DRSP treatment. Estrogen deficiency caused endothelial dysfunction, which was prevented by both treatments. However, the vasodilator response in the E2+DRSP group was significantly higher at the three highest concentrations compared with the OVX group. Reduced ER-α expression in OVX rats was restored by both treatments. Morphometric parameters and oxidative stress were augmented by OVX and reduced by E2 and E2+DRSP treatments. Hormonal therapy with E2 and DRSP may be an important therapeutic option in the prevention of coronary heart disease in hypertensive post-menopausal women.

## Introduction

Coronary heart disease (CHD) is a major cause of morbidity and mortality worldwide (1), and hypertension is established as one of the main risk factors for the development of CHD (2). Prior to menopause, women exhibit lower blood pressure (BP) compared with age-matched men. However, aging and decreased circulating estrogen (E2) levels are accompanied by increased BP, which contributes to a greater prevalence of hypertension and an increased prevalence of CHD during the post-menopausal period (3), suggesting a protective role for E2. Experimental studies analyzing the effects of estrogen replacement therapy in ovariectomized (OVX) rats have demonstrated many beneficial effects on the cardiovascular system. These beneficial effects include the capacity to lower BP in normotensive and hypertensive rats (4-6), reduce oxidative stress (4), prevent endothelial dysfunction, improve endothelium-dependent coronary vascular reactivity, and protect against vascular remodeling in early-stage hypertensive rats (7,8).

However, randomized clinical trials such as the Women's Health Initiative and the Heart and Estrogens/progestin Replacement Study, which analyzed primary and secondary prevention, respectively, have failed to demonstrate such cardiovascular benefits. The conjugated equine E2 plus medroxyprogesterone acetate (MPA) arm of the Women's Health Initiative was prematurely stopped because of the high risk and incidence of CHD, venous thromboembolism, and cancer (9). Nevertheless, although some questions and concerns arose from these studies, the current use of hormone replacement therapy with or without progestins following menopause remains strongly debated.

The undesirable side effects that occur with the use of progestins can manifest because of their interactions with other steroid receptors, such as androgen, estrogen, and glucocorticoid receptors (10). In female swine, progesterone impairs endothelium-dependent relaxation in coronary arteries associated with decreased endothelial nitric oxide synthase (eNOS) immunoreactivity and augmented superoxide production (11). Similarly, MPA, a highly androgenic progestin, reduces endothelial aortic reactivity in aldosterone salt-treated rats after long-term administration. These effects did not occur in groups treated with E2 and drospirenone (DRSP) (12). These results suggest detrimental vascular effects from MPA treatment, and a neutral or beneficial effect from DRSP.

In contrast to other synthetic progestins, DRSP is a progestin that is derived from 17α-spironolactone and has anti-androgenic and anti-aldosterone effects. These properties make DRSP the progestin with the most similar effects to natural progesterone, with the added benefit of a BP-lowering effect (10,13). DRSP has eight times the anti-aldosterone potency of spironolactone, which is a result of its affinity for the mineralocorticoid receptor (MR) (14,15). Previous studies have indicated that combined E2 and DRSP therapy reduces BP in post-menopausal women with stages 1 to 2 hypertension and in young healthy normotensive women (16,17), although other studies found no significant changes (18,19).

Although the vascular benefits of E2 have been described, the effects of combined therapy with E2 and DRSP on endothelium-dependent coronary vascular reactivity are unknown. Because of the BP-lowering characteristic of DRSP, we hypothesized that DRSP does not impair endothelium-mediated vasodilation in the coronary bed of OVX spontaneously hypertensive rats, as already demonstrated with other synthetic analogues of progesterone. Therefore, the aims of this study were to analyze the effects of combined therapy with E2 and DRSP on endothelium-dependent coronary vascular vasodilation in response to bradykinin, as well as on coronary vascular remodeling and oxidative stress in OVX spontaneously hypertensive rats.

## Material and Methods

### Animals

Female, spontaneously hypertensive rats at 12 weeks of age, weighing 160-200 g, were provided by the university facility. All procedures were approved by the Institutional Ethical Committee for Animal Care and Use of the Universidade Federal do Espírito Santo (protocol #023/2012). Experiments were conducted in accordance with the Guide for the Care and Use of Laboratory Animals published by the US National Institutes of Health (NIH Publication, revised 1996). The rats were kept in collective cages with free access to water and standard rat chow (Purina Labina¯, Brazil) under controlled temperature (22-24°C), humidity (40-60%), and light-dark cycle (12-12 h). At the time of ovariectomy, the rats were randomly divided into 4 groups as follows (n=87): sham control (Sham); OVX; OVX treated with estrogen (E2), and OVX treated with estrogen plus DRSP (E2+DRSP).

### Ovariectomy

Ovariectomy was performed under general anesthesia with an intraperitoneal (*ip*) injection of 80 mg/kg ketamine and 12 mg/kg xylazine. A bilateral dorsolateral incision was made through the skin, and the underlying muscle was dissected to locate the ovary and fallopian tube. The tube was ligated with a suture line and the ovary was removed. The muscle and skin were then sutured with an absorbable suture. After the surgery, rats received an injection of antibiotic (2.5% enrofloxacin, 0.1 mL, intramuscularly). Sham rats were incised and sutured, but the ovary was left intact.

### Hormone therapy

E2 therapy was performed by subcutaneous injections (0.1 mL) containing 0.05 mg/kg per day of 17β-estradiol (Sigma, USA) diluted in corn oil, three times per week, as previously described (20). Rats that were treated with DRSP and 17β-estradiol combined therapy received 0.03 and 0.06 mg/kg per day, respectively, administered daily by gavage, starting 7 days after OVX during 6 weeks. The choice of these concentrations was made to mimic the dosage used for hormonal therapy in postmenopausal women. Rats that did not receive any of the therapies had the same volume either injected (corn oil) or gavaged (saline). The effectiveness of the ovariectomy and experimental therapies were assessed by uterine wet weight and the uterine weight to body weight ratio.

### Hemodynamic measurements

At the end of the experimental protocol, rats were anesthetized with ketamine and xylazine (50 and 10mg/kg, *ip*). For direct measurement of systolic BP, diastolic BP, mean arterial pressure, and heart rate, a polyethylene (PE-50) catheter (attached to PE-10 tubing) was inserted into the femoral artery and tunneled to the dorsal neck region. The rats remained in a fasted state until between 7:00 and 8:00 am of the next day to obtain direct measurements of BP in awake and unrestrained rats (TRA021 BP transducer coupled to an ML 110 Amplifier; ADInstruments, Australia). BP and heart rate values were calculated for each rat from continuous recordings averaged over a 30-min period.

### Isolation of coronary arteries

The rats were sacrificed by decapitation. The thoracic cavity was opened, and the heart was removed and placed in cold Krebs-Henseleit buffer solution: 115 mM NaCl, 25 mM NaHCO_3_, 4.7 mM KCl, 1.2 mM MgSO_4_.7H_2_O, 2.5 mM CaCl_2_, 1.2 mM KH_2_PO_4_, 11 mM glucose, and 0.01 mM Na_2_EDTA at pH 7.4 during the dissection procedure. The left anterior descending branch of the left coronary artery and the septal branch were isolated from surrounding ventricular muscle tissue with a dissection microscope (M900; DF Vasconcelos, Brazil) and then snap frozen in liquid nitrogen. The samples were maintained at -80°C until later use.

### Isolated heart preparation (modified Langendorff method)

To assess coronary perfusion pressure (CPP) and endothelium-dependent vasodilation (n=5-6 per group), the rats were anesthetized with chloral hydrate (40 mg/kg, *ip*). The heart was excised and immediately perfused at a constant flow rate. Studies on the coronary vascular bed were performed on whole hearts using a modified Langendorff preparation for perfused isolated hearts as previously described (21). Briefly, using a Langendorff apparatus (Hugo Sachs Electronics, Germany), the isolated hearts were perfused with modified Krebs solution (120 mM NaCl, 1.25 mM CaCl_2_.2H_2_O, 5.4 mM KCl, 2.5 mM MgSO_4_.7H_2_O, 2.0 mM NaH_2_PO_4_.H_2_O, 27.0 mM NaHCO_3_, 1.2 mM Na_2_SO_4_, 0.03 mM EDTA, and 11 mM glucose), equilibrated with a 95% oxygen and 5% carbon dioxide mixture. This was performed at a controlled pressure of 100 mmHg to yield a pH of 7.4, and perfused at a rate of 10 mL/min at 37°C with a peristaltic pump (MS-Reglo 4 channels; Hugo Sachs Electronics). A fluid-filled balloon was introduced into the left ventricle through a steel cannula with a latex balloon and connected to a TPS-2 Statham transducer (Incor, Brazil) to measure the isovolumetric force. The balloon was pressurized with a spindle syringe until it reached a preload of 10 mmHg. CPP was monitored with a TPS-2 Statham transducer connected to a sidearm of the aortic perfusion catheter. After a 40-min stabilization period, baseline CPP was measured. Endothelium-dependent vasodilation was randomly analyzed in the coronary arterial bed by bolus administration of 0.1 mL bradykinin (10^-10^ to 10^-6^ M).

### Western blotting

The coronary arteries were pooled with frozen tissue from 3 rats (considered as n=1), and the total number of pooled samples per group was n=4. The samples were homogenized in lysis buffer containing 150 mM NaCl, 50 mM Tris-HCl, 5 mM EDTA, 2 mM Na, and 1 mM MgCl_2_ plus protease inhibitors (Sigma Fast, USA). The protein concentration was determined by the Lowry method (22) and bovine serum albumin was used as the standard. Equal amounts of protein were denatured and separated by 10% sodium dodecyl sulfate-polyacrylamide gel electrophoresis and transferred onto a polyvinylidene fluoride membrane (Millipore, Germany). The membranes were blocked with 5% bovine serum albumin at room temperature in TBS buffer plus Tween 20 (0.1%) before incubation with monoclonal anti-mouse for eNOS (1:1500; BD Biosciences, USA), polyclonal anti-mouse for estrogen receptor alpha (ER-α) (1:500; Santa Cruz Biotechnology, USA), and polyclonal anti-mouse for β-actin (1:1500; Sigma). After washing, the membranes were incubated with an alkaline phosphatase conjugated anti-mouse IgG (1:3000; Abcam Inc., USA). The bands were visualized using an NBT/BCIP system (nitroblue tetrazolium/5-bromo-4-chloro-3-indolyl-1-phosphate; Invitrogen, USA) and quantified using ImageJ software (National Institutes of Health, USA).

### Histomorphometry

After perfusion fixation, the septal branch of the coronary artery was dissected free of surrounding tissue as described above. Frozen samples (n=4) from the coronary artery in OCT compound were cut into 8-μm-thick sections and mounted on gelatin coated glass slides. The sections were stained with hematoxylin and eosin. The histomorphometric image analysis system consisted of a digital camera (Axio-Cam ERc 5S, Zeiss, Germany) coupled to a light microscope (Olympus AX70; Olympus, USA). High-resolution images (2048×1536 pixels buffer) were captured using a Carl Zeiss AxioVision Rel. 4.8 (Germany). Photomicrographs were obtained using a 20× objective, and the total vascular area, lumen area, vessel wall area, and wall/lumen area were calculated with the area measure tool of AxioVision Rel. 4.8.

### Detection of superoxide production (dihydroethidium fluorescence)

Unfixed frozen sections from the coronary arteries (n=4) were cut into 8-μm-thick sections and mounted on gelatin-coated glass slides. To detect superoxide, samples were incubated with the oxidative fluorescent dye dihydroethidium (DHE, 2 μmol/L) in a modified Krebs's solution (containing 20 mM HEPES) housed in a light-protected humidified chamber at 37°C for 30 min. The intensity of fluorescence was detected at 585 nm and quantified in the tissue sections using a confocal fluorescent microscope (Leica DM 2500 TI; Nikon Instruments Inc., USA) by an investigator who was blind to the experimental protocol.

### Statistical analysis

Data are reported as means±SE. One- and two-way ANOVAs were used when appropriate. Tukey's *post hoc* test was used for multiple comparisons. Differences were considered to be significant when P<0.05.

## Results

### Surgery and efficacy of hormonal therapy

To verify the estrogenic status of the rats, following sacrifice, the uterus was removed and weighed dry. In the OVX group, the weight of the uterus and the uterus to body weight ratio were lower compared with those in the Sham group (both P<0.05). Both treatments prevented atrophy of the uterus, and the weight of the uterus and the uterus to body weight ratio were significantly greater with both treatments compared with OVX (P<0.05), indicating the efficacy of hormonal therapy (Table 1).

**Table:**
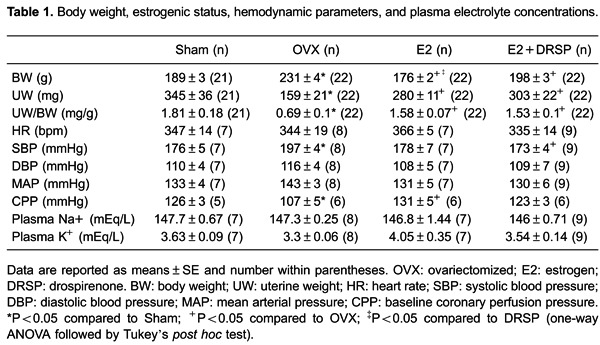

### Hemodynamic measurements and plasma electrolyte concentrations

Systolic BP in hypertensive rats after ovariectomy was significantly higher than that in the Sham group (P<0.05). Moreover, both treatments restored systolic BP to control levels, but only the E2+DRSP group reached statistical significance (P<0.05 *vs* OVX). However, heart rate, diastolic BP, and mean arterial pressure were not significantly different among the groups (Table 1). There were no significant differences in plasma sodium and potassium concentrations among the groups (Table 1).

### Baseline CPP and endothelium-dependent vasodilator response to bradykinin

**Figure 1:**
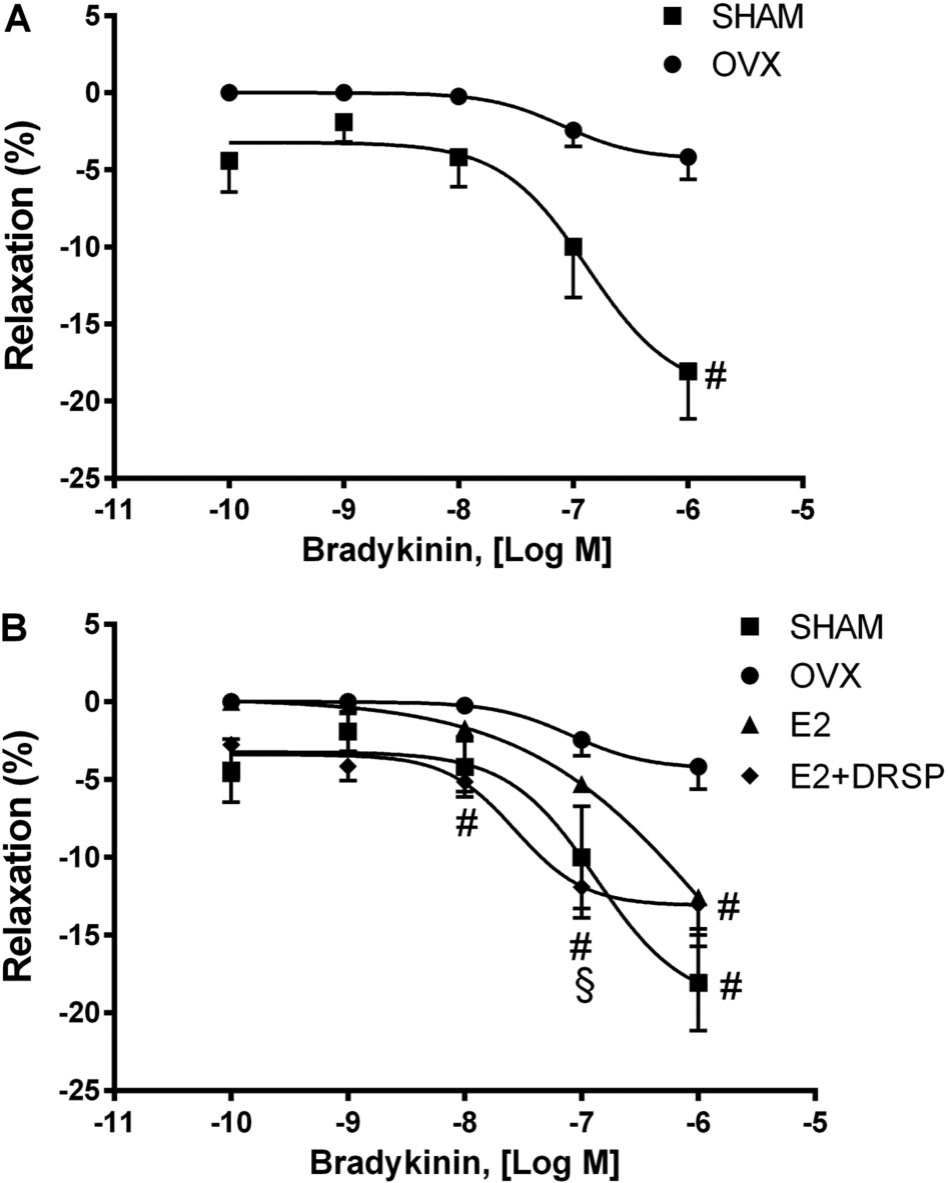
Endothelium-dependent coronary vasodilation with bradykinin. Vasodilation caused by estrogen deficiency (*A*). Effects of E2 and E2+DRSP treatments on coronary vascular function (*B*) in ovariectomized spontaneously hypertensive rats. Data are reported as means±SE (n=5-6 per group). OVX: ovariectomized; E2: estrogen; DRSP: drospirenone. ^#^P<0.05 compared to OVX, ^§^P<0.05 compared to E2 (two-way ANOVA followed by Tukey's *post hoc* test).

Baseline CPP was significantly reduced in the OVX group compared with the Sham group (P<0.05). Endothelium-dependent coronary vasodilation in response to bradykinin showed an impaired response in the OVX group compared with the Sham group (P<0.05; Figure 1A). However, this impairment was restored by estradiol treatment alone or in combination with DRSP compared with OVX rats (P<0.05; Figure 1B). Moreover, combined therapy with DRSP improved the effects of E2 because the endothelium-dependent response was significantly higher at the three highest concentrations compared with the OVX group, and was significantly higher compared with the E2 group at the concentration of 10^-7^ M (P<0.05).

### Coronary expression of eNOS and ER-α

**Figure 2:**
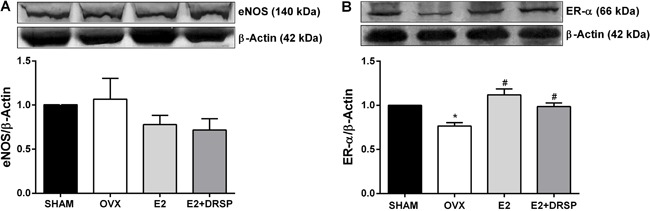
Protein expression of endothelial nitric oxide synthase (eNOS) (*A*) and estrogen receptor alpha (ER-α) (*B*) in coronary arteries from ovariectomized spontaneously hypertensive rats. Data are reported as means±SE (n=4 per group). OVX: ovariectomized; E2: estrogen; DRSP: drospirenone. *P<0.05 compared to Sham, ^#^P<0.05 compared to OVX (one-way ANOVA followed by Tukey's *post hoc*test).

Protein expression of eNOS did not differ among the groups (Figure 2A). We evaluated the effects of ovariectomy and experimental treatments on ER-α protein expression because of the known effects of ER-α activation on enhancement of eNOS activity. Ovariectomy led to a significant reduction in ER-α protein expression (P<0.05), which was restored by both treatments (both P<0.05 *vs* OVX; Figure 2B).

### Morphometric assessment

**Figure 3:**
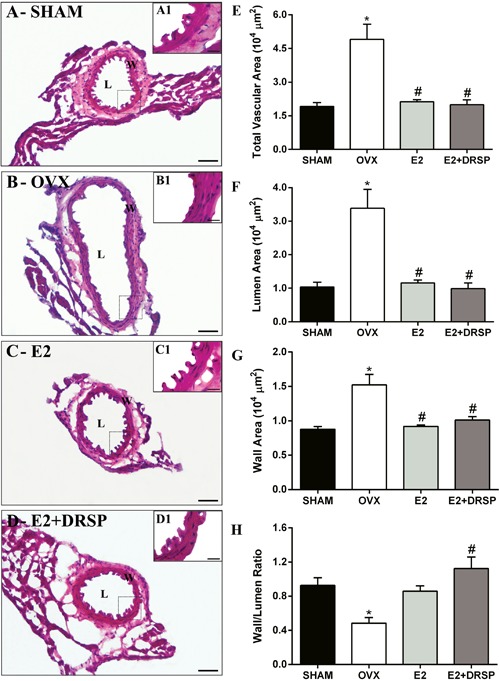
Histomorphometric analysis of coronary arteries from ovariectomized spontaneously hypertensive rats. Representative images of histological slices of the coronary arteries: SHAM (A and A1), OVX (B and B1), E2 (C and C1), DRSP (D and D1) groups. Total vascular area (*E*), lumen area (*F*), vessel wall area (*G*), and the wall to lumen ratio (*H*) are reported as means±SE (n=4 per group). OVX: ovariectomized; E2: estrogen; DRSP: drospirenone; L: vessel lumen; W: vessel wall; total vascular area: L+W area. *P<0.05 compared to Sham, ^#^P<0.05 compared to OVX (one-way ANOVA followed by Tukey's *post hoc* test). Bars: 50 and 20 µm.

We performed morphometric analysis to analyze the effects of ovariectomy and experimental treatments on vascular remodeling in coronary arteries (Figure 3). The total vascular area, vessel wall area, and lumen area were augmented in the OVX group compared with the Sham group (P<0.05). However, E2 and E2+DRSP treatments prevented these changes, and these areas were maintained at the same level as in the Sham group and reduced compared with the OVX group (P<0.05). The wall to lumen ratio was reduced in the OVX group compared with the Sham group (P<0.05) because of higher vessel wall and lumen areas. Only E2+DRSP treatment resulted in a higher wall to lumen ratio compared with the OVX group (P<0.05).

### DHE fluorescence

**Figure 4:**
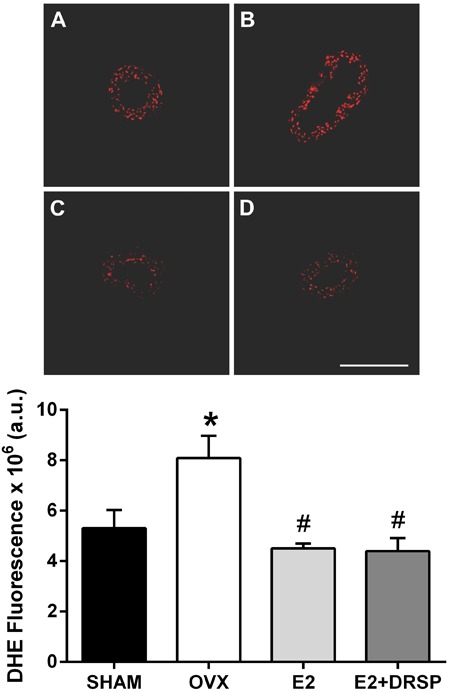
Analysis of oxidative stress in coronary arteries from ovariectomized spontaneously hypertensive rats by dihydroethidium fluorescence (DHE). Data are reported as means±SE (n=4 per group). Representative images of Sham (*A*), OVX (*B*), E2 (*C*) and E2+DRSP (*D*) groups are shown. OVX: ovariectomized; E2: estrogen; DRSP: drospirenone.*P<0.05 compared to Sham, ^#^P<0.05 compared to OVX (one-way ANOVA followed by Tukey's *post hoc*test). Bar: 200 µm. a.u.: arbitrary units.

To evaluate the effects of treatments on vascular oxidative stress *in situ*, we detected superoxide anion production by the DHE method (Figure 4). Formation of superoxide anion was increased in the coronary arteries of OVX rats compared with Sham group (P<0.05), which could contribute to oxidative stress and reduced NO bioavailability. Estradiol alone or in combination with DRSP was able to reduce superoxide formation (P<0.05) to a similar level observed in the Sham group.

## Discussion

The main findings of this study were as follows: *i*) E2 therapy and combined therapy with E2 and DRSP restored endothelial dysfunction caused by estrogen deficiency in hypertensive rats; *ii*) E2+DRSP improved endothelium-dependent coronary vascular reactivity, and *iii*) DRSP did not impair the estrogenic actions related to prevention of remodeling and vascular oxidative stress.

A decline in plasma E2 concentrations following OVX can cause elevated BP in normotensive rats and exacerbate the hypertensive framework of hypertensive animals (4-6),^23^ as observed in our study. This finding suggests an important role for E2 on regulation of BP. Based on this finding and the fact that hypertension is an independent risk factor for CHD, women in the post-menopausal period are much more prone to develop this disease, mainly when they are hypertensive. Therefore, determining therapies that can contribute to improvement of this condition, especially considering the particular conditions of the post-menopausal period, is extremely important.

A previous clinical study evaluated the antihypertensive effects of DRSP in post-menopausal women with stages 1 to 2 hypertension (16). This study showed that DRSP at concentrations of 2 and 3 mg conjugated with E2 significantly reduced BP without altering serum potassium concentrations and increasing serum aldosterone concentrations. Similar results were found in another study where 2 mg of DRSP caused a reduction in both systolic and diastolic BP (24).

The BP-lowering effect of DRSP is related to its anti-aldosterone property, which is characterized by inhibition of the MR. This feature has fundamental importance in the regulation of BP. This is because in addition to inhibiting salt and water retention in the kidneys, MR directly regulates BP in vascular smooth muscle cells (25). McCurley et al. (25) showed that systolic BP in mice with specific deletion of MR in vascular smooth muscle cells was significantly lower compared with that in control animals. This lower systolic BP was maintained, even when the mice aged and were stimulated with angiotensin II infusion. Therefore, the BP-lowering effects of DRSP may be of great therapeutic value for hypertensive women in the post-menopausal period.

In the current study, although CPP was decreased in OVX rats, endothelium-dependent vasodilation in response to bradykinin was extremely reduced in this group. This finding clearly indicates a worsening of endothelial function associated with estrogen deficiency in spontaneously hypertensive rats. This finding was confirmed by morphometric images, where a lack of the vascular endothelial layer in the OVX group (Figure 3B) was observed. Moreover, the lack of E2 increased vascular oxidative stress, as demonstrated by DHE staining. Indeed, OVX can increase oxidative stress independently of blood pressure levels. A reduction in levels of E2, which is a known antioxidant hormone, increases the activity of pro-oxidant generating pathways, such as NADPH oxidase (26), and decreases the protein expression of antioxidant enzymes (7,27). Therefore, decreased bioavailability of nitric oxide (NO) can occur because this molecule quickly reacts with superoxide, forming peroxynitrite. Peroxynitrite is primarily responsible for the uncoupling of eNOS due to oxidation of the enzyme co-factor tetrahydrobiopterin (28). Furthermore, peroxynitrite can reduce vascular relaxation mediated by endothelium-derived hyperpolarizing factors through inhibition of large conductance of calcium-activated potassium channels (29).

In our study, deterioration in endothelial function was prevented by E2 therapy and by combined therapy with E2+DRSP. Other synthetic progestins that are used in post-menopausal hormonal therapies, such as MPA, can impair or antagonize the beneficial actions of estradiol on vascular function (12). In the present study, combined therapy with E2+DRSP in spontaneously hypertensive rats demonstrated a more pronounced vasodilatory response because it was significantly greater at the three highest concentrations evaluated compared with OVX rats, suggesting that this progestin improves E2 effects on coronary function. Human studies assessing flow-mediated dilation demonstrated that combined therapy can improve endothelium-dependent vascular function in young women and in healthy women in the post-menopausal period (30,31).

Based on these previous findings, this study aimed to evaluate the endothelium-dependent pathway. We analyzed protein expression of the eNOS enzyme to verify if the effects of the therapies were associated with enhancement of this enzyme. However, we did not detect any differences in eNOS expression among the groups. This finding suggests that neither E2 nor E2+DRSP can alter vascular expression of this enzyme. These results are in contrast with previous studies, which reported an increase in the expression of eNOS after OVX treatment in cerebral blood vessels and in culture of human endothelial cells (32,33). Nevertheless, not all studies have reported an increase in eNOS after OVX and E2 treatment. A previous study from our laboratory also demonstrated no change in eNOS protein expression in coronary arteries after E2 treatment in OVX rats (7), and another study showed a reduction in eNOS expression in the aorta (26). However, in the present study, decreased expression of ER-α with OVX was restored by both treatments. Although we did not find any differences in eNOS expression among the groups, the increase in ER-α expression could explain, in part, the improved endothelium-dependent reactivity in the treated groups. This is mainly because the binding of E2 to this receptor can increase enzymatic activity of eNOS, augmenting NO release as previously reported by Chen et al. (34). Another important factor in our study was the reduction in vascular oxidative stress by the treatments because an increase in reactive oxygen species can reduce the bioavailability of NO, as mentioned above.

Our results of protein expression, histomorphometry, and the reduction in oxidative stress appear to be more related to the effects mediated by E2 than those by DRSP. We did not detect any differences between the E2 and E2+DRSP groups on the parameters cited above, demonstrating a neutral effect of DRSP. However, our results are important because other synthetic progestins reduce endothelial expression of eNOS, which is associated with decreased NO production (35,36). This phenomenon possibly culminates with degradation in vascular function. Accordingly, Arias-Loza et al. (12) demonstrated enhancement of vascular contraction in response to phenylephrine, as well as a reduction in endothelium-dependent and independent vascular relaxation of aortic rings when exposed to MPA.

According to previous studies, we suggest that factors other than NO may be associated with improvement of endothelium-mediated vasodilation in the E2+DRSP group. DRSP can increase the enzymatic activity of eNOS via the progesterone receptor, and in conjunction with E2, it can increase NO production (33). Moreover, inhibition of MR by DRSP causes augmentation of serum aldosterone concentrations (16,37). Aldosterone activates rapid responses in vascular reactivity, which are not consistent with genomic activation (38). Gros et al. (39) showed in rat aortic rings that aldosterone significantly reduced constriction induced by phenylephrine. Another study reported that aldosterone acts as an endothelium-dependent vasodilator in rat cerebral and mesenteric arteries through NO (40).

Therefore, taking into consideration inhibition of MR, based on our findings of an increase in serum aldosterone concentrations and a functional and intact endothelium, these factors could be possible mechanisms involved in improvement of endothelial vasodilation due to E2+DRSP treatment. However, establishment of a causal relationship between these factors in hypertensive OVX rats and endothelium-dependent coronary vascular reactivity remains to be determined. Further studies on this issue are necessary.

This study showed that E2+DRSP therapy, beyond the known effects on reduction of BP, can improve endothelium-mediated vasodilation in the coronary bed and did not impair estrogenic effects on coronary vascular remodeling and oxidative stress in OVX spontaneously hypertensive rats. These results are of great importance considering that DRSP simultaneously acts in a similar manner as progesterone to protect against hyperplasia of the endometrium, and it can improve the beneficial cardiovascular effects observed in experimental models with estrogenic therapy. Therefore, hormonal therapy with E2 and DRSP may be an important therapeutic option in the prevention of coronary heart disease in hypertensive post-menopausal women.
